# Digital badges in academia: An educational tool for the clinical research coordinator

**DOI:** 10.1017/cts.2024.490

**Published:** 2024-02-28

**Authors:** Barbara DeMarco, Yasheca Ebanks, Barbara Tafuto

**Affiliations:** 1 Rutgers School of Health Professions, Newark, NJ, USA; 2 New Jersey Alliance for Clinical and Translational Science, Newark, NJ, USA

**Keywords:** Badging, clinical research coordinator, competencies, micro-credential, workforce development

## Abstract

Digital badges can provide condensed competency-based knowledge enabling individuals a chance to explore specialized careers in clinical research. A digital badge can be an efficient pathway to introduce clinical research job roles and educate a larger diverse workforce for clinical research coordinator positions at AMCs. The New Jersey Alliance for Clinical and Translational Science (NJ ACTS) developed a digital badge with potential to broaden exposure to training opportunities for CRCs and improve their prospects for a career at Rutgers. This paper describes the development of a digital badge introducing individuals to the clinical research profession, especially for those who aspire to become a CRC. The badge was designed to include five domains (Scientific Concepts and Research Design, Ethical and Participant Safety Considerations, Clinical Study Operations and Site Management, and Data Management and Informatics). Participants assessed the badge for accuracy and presentation level. The results demonstrated that the competencies were met, and content was appropriate for someone with limited knowledge of clinical research. Survey results along with the Difficulty Index and Discrimination Index calculated for quiz questions supported the badge rank as foundational. Research is ongoing to evaluate the value of the badge to job acquisition, performance, and career growth.

## Introduction

A digital badge is a visual representation of a validated learned skill or competency [1]. A digital badge focuses on one distinct topic as opposed to an academic course which may cover several topics and skills addressed over a semester [2]. The competency-based component of a digital badge allows for opportunities to earn multiple badges that can be taken to increase a competency level sequentially to form a micro-credential.

Traditionally a mainstay of scouting and the military, the modern form of badging can be presented in a digital format in email signature lines and LinkedIn profiles. The concept of digital badging has recently become of greater interest among institutions of higher education [3]. Badging has the potential to be an effective tool for recruitment into academia by serving as a stepping-stone to more comprehensive educational programs offered by the institution backing the badge. For this reason, academia has been increasingly offering digital badges alongside more traditional certificates and degrees. For learners, this trend of digital badges in academia can open the doors to a more focused learning platform since users can earn a university-backed credential without the traditional time commitment, financial investment, or time-related challenges [4]. Users in this format have the freedom to pick and choose a badging program that matches their educational needs and aligns with their professional goals. They are not required to enroll in the university or take any additional courses. The fact that digital badges can be acquired at an exponentially lower cost option to standard course credits opens doors to users unable to earn standard degrees.

Digital badges are not foreign to medical or health-related education even though barriers to acquiring the training and education needed for a health-related workforce can be challenging. Access to medical or health-related programs and degrees is not available at every institution of higher education. Needed competencies for niche health-related workforce roles like Clinical Research Coordinators can be difficult to acquire [5]. Competencies such as subject recruitment, enrollment, and consenting are common to this workforce. Administrative competencies including preparing for audits, ensuring regulatory document compliance, reporting adverse events, and maintaining research standards are just a few additional competencies that make the CRC’s role unique to a clinical research team. The fact that digital badges can verify specific skill sets to employers, allows applicants to present the badge as evidence of CRC job-related competencies [5]. Badging represents a clear, and non-disputable documentation of skills and expert knowledge.

The New Jersey Alliance for Clinical Translational Science (NJ ACTS), a National Center for the Advancement of Clinical and Translational Science’s CTSA hub since 2019, established a Workforce Development core to address barriers and challenges to training and educating clinical research professionals. To provide the evidence-based competencies needed to be a CRC, NJ ACTS designed and developed a Clinical Research Coordinator Digital Badge (CRC Badge). The NJ ACTS CRC Badge was created to build clinical research-related skills to support learners interested in entering or advancing in the role of the CRC.

The purpose of this Special Communication is to describe the development of a digital badge designed to introduce individuals to the CRC position.

## Methods

### Content development

Seven clinical research professional experts from Rutgers Health with a combined 132 years of experience in clinical and translational science at an academic medical center (AMC) were assembled to identify key competencies needed to run trials. These individuals included: (1) Executive Director of the Clinical Trials Office; (2) Workforce Development Core Leads; (3) Manager of Quality Assurance and Quality Control at NJ ACTS; (4) Associate Director of Clinical Trials Administration at the Cancer Institute of New Jersey; (5) Nurse Manager for Clinical Research at Robert Wood Johnson Medical School; and (6) Operations Manager at the Environmental and Occupational Health and Science Institute.

Incorporating the Delphi Method for collecting perspectives, this panel of experts reviewed the Joint Taskforce for Clinical Trial Competencies (JTF) as the foundation to identify relevant knowledge domains for a Level 1 CRC Position. The group chose 6 of the 8 domains; (1) Scientific Concepts and Research Design, (2) Ethical and Participant Safety Considerations, (3) Investigational Products Development and Regulation, (4) Clinical Study Operations, (5) Study and Site Management, and (6) Data Management and Informatics [6]. The JTF domains relating to Leadership & Professionalism and Communications & Teamwork were excluded since it was felt that these competencies would be more difficult to assess in the proposed badge format. Additionally, 2 JTF domains relating to clinical study operations and site management were merged since these competencies included tasks that may occur simultaneously at the site and with the sponsor. The experts then listed competencies needed by a CRC under each domain that matched an entry-level job role at Rutgers. They identified 5–9 foundational or level 1 competencies per domain that were relevant to this AMC and then proceeded to write course content directed toward teaching these competencies (Table 1).

**Table 1.** Module competencies

**Module one competencies: scientific concepts and research design**
1. Review the phases of drug development and be able to explain the process to subjects
2. Define Adverse Drug Reactions and develop a series of questions to help identify their presence
3. Describe the process used to report Adverse Drug Reactions outside of a trial
4. Discuss the phases of pharmacokinetics and be able to demonstrate to a subject a drug’s path through the body from ingestion to elimination
5. Describe drug-receptor relationships and dose-response curves and apply these concepts to drug safety
6. Summarize commonly used study designs (case series, case-control, cross-sectional, cohort, experimental, qualitative, and correlation studies)
7. Define and apply basic statistical terminology (p-value, sample size, bias, randomization, stratification, blinding)
8. Interpret study results by considering Tests of Comparison (Parametric and Nonparametric) and Association (Regression Analysis)
9. Explain Evidence-Based Medicine
**Module two competencies ethical and participant safety considerations**
1. Explain the historical evolution of ethics and human subjects protection in clinical research
2. Describe key legislation and reasons for enactment for the following acts: Federal Food & Drug Act (1906); Food, Drug & Cosmetic Act (1938); Nuremberg Code (1947); Kefauver-Harris Amendments (1962); Declaration of Helsinki (1964); Belmont Report (1979); ICH (1997); HIPAA (1996)
3. Summarize the regulatory requirements for protection of human subjects as described in 21CFR Part 46, Part 50, and Part 56
4. Describe the ethical issues involved in the recruitment of vulnerable research participants
5. Describe the mission, function, and procedures of the Institutional Review Board
6. Describe the roles and responsibility of the Data and Safety Monitoring Board
7. Discuss HIP authorization process relating to informed consent
8. Apply the process of consenting non-english speaking participants, vulnerable, subjects and special populations and explain the reasons for consent to subjects
9. Determine if a Serious Adverse Event has occurred and explain the reporting timelines
**Module three competencies investigational products development and regulation**
1. Describe the regulatory responsibilities of the various institutions participating in the investigational product development process
2. Determine the regulations that apply to research and use of investigational products
3. Describe the Investigational New Drug Application and the New Drug Application
4. Describe phases of drug development in detail
5. Explain the purpose of a clinical trial with respect to drug development
**Module four competencies clinical study operations and site management**
1. Distinguish the stages of clinical research and key milestones from protocol concept to final results
2. Identify the diverse regulations associated with the different stages of protocol concept to final results
3. Recognize the key interdisciplinary players
4. Differentiate between the responsibilities of a Sponsor and the Investigator and clinical research site
5. Identify the lifecycle of a clinical trial at a research site
6. Describe the elements of conducting an informed consent
7. Recognize the sections of a Regulatory Binder outlined by Good Clinical Practice
8. Explain the differences in billable procedures verses research procedures
9. Describe the goals of a study audit
**Module five data management and informatics**
1. Discuss the function of a clinical trial management system
2. Explain data privacy regulations
3. Explain good source documentation
4. Explain difference between Adverse Event, Adverse Drug Reaction, and Serious Adverse Event
5. Report all adverse events to the sponsor within the correct time period
6. Explain the difference between Adverse Event, Adverse Drug Reaction, and Serious Adverse Event with regards to data management
7. Describe methods for assuring data quality

### Badging course design

The badging course included 5 educational modules housed within Canvas, our learning management system, each containing video lectures, handouts, a discussion forum, and a corresponding 25-question quiz. The discussion forum served as a repository for questions on content or logistics and was monitored by the team’s project manager.

### Pilot testing

To assess the badging course design, implementation, and presentation level (level of difficulty), participants were recruited for pilot testing in 2 phases. Pilot 1 was conducted in July 2022 to review for typographical and content errors, course performance and navigation, as well as overall quality. Pilot 2 was conducted in January 2023 and included updates and recommendations from Pilot 1. The participants were required to complete the modules asynchronously but sequentially and could not progress to the next module until the quiz for that module was completed with a score of 90%. If a participant failed a quiz after three attempts, the plan was to remove the individual from the badging course and enroll them in a future offering to start over.

Data collection points from the pilot testing process included assessing: (1) course difficulty level - based on quiz scores and the questions’ Difficulty Index; and (2) completeness of course competencies from a participant exit survey.

## Results

**Figure 1.**
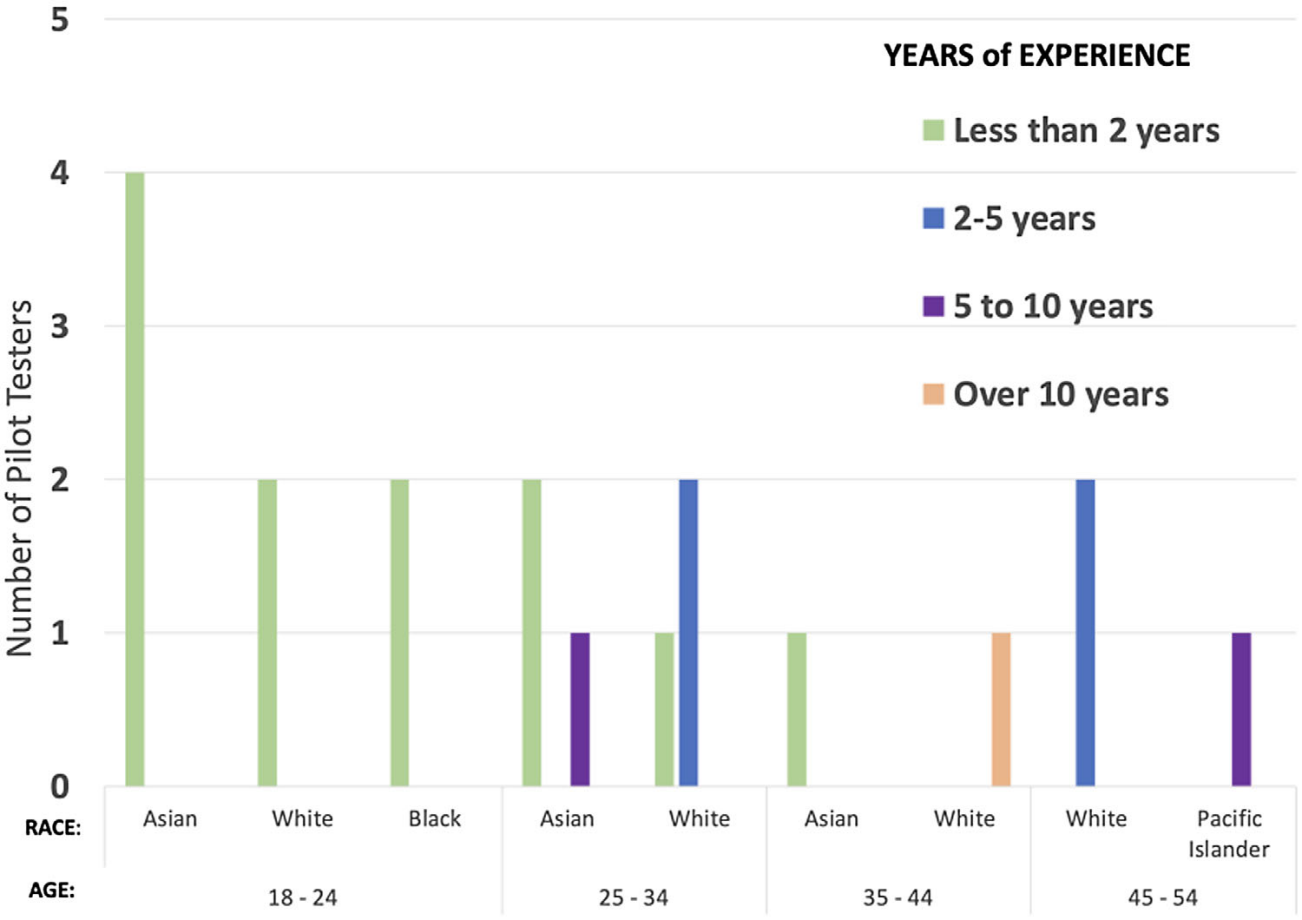
Participant years in the field by age and race.

Nine participants were originally referred to Pilot 1 by principal investigators, clinical research administrators, and senior management at a clinical trials solution company that provides staffing solutions for Rutgers University clinical research units. Seven of the 9 testers completed the badging course in its entirety. One participant dropped out before beginning the module. One additional individual dropped out before completing the last module due to personal time commitments. Participants for Phase 2 were recruited through advertisements within the NJ ACTS newsletter and distribution of email flyers to marketing and communications individuals within the medical and health-related schools. Twenty-three participants enrolled in the badging course for Phase 2. However, of the 23 volunteers, 11 did not complete the course, resulting in a total of 19 participants for the pilot phase 1 and 2. Of these 19 volunteers, 4 were male and 15 were female. Eight of the individuals identified as white and another 8 identified as Asian. There were 2 Black/African Americans and 1 individual who identified as Native Hawaiian or Other Pacific Islander. They ranged from no degree (undergraduate students) to having earned a terminal degree and with varying years of experience as a CRC (Fig. 1).

### Participants’ skill level

To determine if the badging course was geared towards foundational versus advanced CRCs, participants with expertise along the continuum between no experience and those with advanced skills had to be recruited. Since participants who volunteered in phase 1 were invited testers, this approach was reserved for phase 2 volunteers who self-selected to participate in this pilot project. Therefore, participants in phase 2 were asked to report their level of expertise in the following areas: data collection and management, enrollment and recruitment, and regulatory activities. Three participants had “no previous experience” in these areas or possessed a mix of “no experience” in some categories and “fundamental expertise” in others. Four participants reported being skilled and advanced in the same areas. The remaining individuals had a combination of fundamental, skilled, and advanced expertise. Figure 1 presents the number of years as a CRC for participants in both phases by age and race.

### Assessing quiz questions

**Figure 2.**
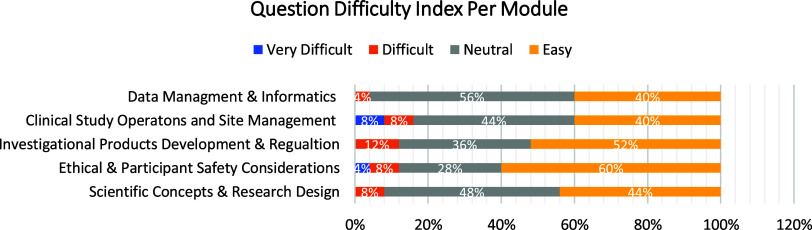
Question difficulty.

Since the badge was geared towards individuals with limited experience it was necessary to determine whether the questions were also constructed on a foundational level. Therefore, an item analysis for each quiz question was performed through Canvas to determine the “difficulty index” (DI). The “difficulty index” refers to the percent of participants who scored correctly on an item [7]. This score ranged from 0.0 to 1.0 with the more difficult questions scoring low, and easy questions scoring higher. The DI measures question difficulty but also is used to identify questions that were poorly written. Based on their DI score, questions were classified into four categories, “easy” (0.9–1.0), “neutral” (0.6–0.89), “Difficult” (0.3–0.59), and “Very Difficult” (< 0.29), and percentages of each question type was determined for all the modules [7] (Fig. 2). Out of 125 questions across all modules, 47% had a DI score that placed them in the “easy” range and 42% tested in the “neutral” category.

The Discrimination Index (D) is another metric used to assess multiple-choice questions. This index determines how well a question “discriminates” between high-performing participants and lower-performing participants [8]. Questions in which high-performing participants answer correctly and lower-performing participants answer incorrectly would have a high “D.” But if the opposite were true and a high-performing group answered incorrectly but the lower-performing students on average answered correctly, the value for “D” would be a negative number. A highly discriminatory question is considered + 0.25 or above. A score of 0.0 indicates that just as many higher-performing students as lower-performing students answered correctly. A negative number indicates that the higher-performing group scored lower than the lower-performing group on an item and should be rewritten. Out of 125 quiz questions in our badging course, four questions with *D* values of −0.01, −0.23, −0.01, and −0.03, had to be rewritten.

### Survey results

The participants successful in earning the badge (*n* = 19) completed a survey providing feedback on course mechanics, content accuracy, question clarity, time to completion, competencies met, and whether the content was engaging. Except for the open-ended questions, all questions were scored using a Likert scale: Strongly Agree, Agree, Neutral, Disagree, and Strongly Disagree or “Yes” or “No.” See Table 2 for survey results.

**Table 2.** Exit survey questions

Survey question	Results
Module Navigation was easy	Strongly agree or agree: 100%
Content was accurate	Yes: 95%
	No: 5%
Questions were clearly written	Strongly agree or agree: 67%
	Neutral: 11%
	Disagree: 16%
Average time to complete all modules	13.25 hours with a range of 4.25 hours–23 hours.
Competencies met	Yes: 100%
Content was engaging	Strongly agree or agree: 78%
	Neutral: 5%
	Disagree: 17%
Reason for participation (check all that apply)	Enter the field: 56%
	Increase knowledge: 89%
	Make a career shift: 11%
	Add badge to resume: 67%
	To replace formal education: 0%

### Badging course non-completers

All Pilot 1 participants were allotted 4 weeks to earn the badge. However, on the recommendation from Pilot 1, Pilot 2 participants were granted an additional 2 weeks. Despite the increase in time, 48% (*n* = 11) of participants either did not start the course or were non-completers. The main reason for not starting or not completing the course was the time commitment.

## Discussion

Badges have transcended history from tangible symbols used in the military, sports, and entertainment to digital badges used in business and now academia. However, no matter whether they are physical or digital symbols, they represent motivation, acquired learning, and belonging to a specialized group.

Badges also provide alternative pathways to achievement when a degree may be unattainable for certain population groups. Born from the pandemic, new learners desire a more granular focus as well as an affordable alternative to higher education and a compact program that can be completed at their own pace [9]. Badges are unlikely to eliminate the need for degree offerings but may be a gateway into a program or job by offering foundational knowledge [10,11]. Hence, that was the goal behind developing this CRC Level 1 Badge.

Critical to this project was to evaluate whether the badge was presented on a foundational level. To achieve this goal, perspectives from advanced CRCs and those naïve to the role were needed for pilot testing. Having testers with varying experiences ranging from “no experience” to “more than 10 years” improved the accuracy of the Discrimination and Difficulty Indices. If all volunteers were at the advanced level, the item analysis would not discriminate between easy and more difficult questions.

Although the volunteers were never asked to specifically identify the content as either foundational or advanced (which would be an opinion), the Difficulty Index indicated that the majority of quiz questions tested within the “easy” or “neutral” range inferring that the participants understood the material including those with limited exposure to clinical research. Experienced CRCs provided feedback on content accuracy and whether the course covered the competencies, while those individuals with either “No experience” or “Foundational expertise” commented on the course and test question clarity. Feedback obtained from the volunteers indicated that the content was clearly presented and accurate, the course was easy to navigate, and all course competencies were met, plus the item analysis of quiz questions indicated that the badge was designed at a foundational level.

When this project was conceived, the goal was to create a vehicle to introduce individuals from diverse health professions or science fields to clinical research by the creation of a digital badge providing foundational education. Since the initial pilot groups described above, we have conducted three additional badging courses and awarded a total of 47 digital badges representing a 54% completion rate. Further courses have been scheduled through 2024, with some already at capacity with waiting lists. Additionally, the badge will be piloted by first-year medical students and individuals in the MD/PhD program to determine if some of the modules would satisfy new competencies in medical education for clinical research.

The process for conducting the badging course is continuously undergoing improvements. To address the non-completion rate participants are now being charged $35.00 of which $30.00 is refundable upon completion of the badge. Returned funding has been a motivating factor toward improving completion rates. Additionally, the time commitment to earn the badge is thoroughly explained to everyone who inquires about the course.

In addition to process improvements, evaluation is ongoing to establish the usefulness of the badge in facilitating the onboarding process of new hires at Rutgers Cancer Institute of New Jersey. Participants will be monitored to determine if the badge translates to improved job performance. All participants will be surveyed at 6- and 12-months post course completion to retrospectively query if they thought earning the badge was valuable. Additionally, their employer will provide feedback comparing the performance of new hires who earned the badge, with those who did not benefit by enrolling in the course.

## Conclusion

We described the development of a digital badge to introduce the foundational knowledge necessary to become a clinical research coordinator at an AMC. Our team of experts in clinical research outlined level one competencies associated with the entry-level position and then subsequently developed an online, asynchronous, five-module badging course addressing those competencies. The badge then underwent 2 pilot testing phases to identify any content or clarity issues. The Difficulty Index and Discrimination Index, plus participants feedback, acknowledged the desired competency level. Research is ongoing to determine the value of the badge on recruitment into the field and on job performance. While the team acknowledges that the concepts covered in the course are not comprehensive enough to enable someone new to the CRC role to immediately practice independently, it should prove to be a useful tool to attract individuals to the field of clinical research and to shorten the onboarding process.

## References

[1] Peisachovich EH , Dubrowski A , Da Silva C , Kapralos B , Klein JE , Rahmanov Z. Using simulation-based methods to support demonstration of competencies required by micro-credential courses. Cureus. 2021;13(8):e16908. doi: 10.7759/cureus.16908. 34513481 PMC8418224

[2] Abramovich S , Schunn C , Higashi RM. Are badges useful in education? It depends upon the type of badge and expertise of learner. Educ Technol Res Dev. 2013;61(2):217–232.

[3] Wilson B , Gasell C , Ozyer A , Scrogan L. Adopting digital badges in higher education: scoping the territory. In: Ifenthaler D , Mah DK , eds. Foundation of Digital Badges and Micro-Credentials. Switzerland: Springer International; 2016:163–177.

[4] Ralston SJ. Higher education’s microcredentialing craze: a postdigital-deweyan critique. Postdigit Sci Educ. 2021;3(1):83–101. doi: 10.1007/s42438-020-00121-8. PMC7235178 40476912

[5] Noyes JAW , Johnson PM , Carbonneau KJ. A systematic review of digital badges in health care education. Med Educ Rev. 2020;54:600–615. 10.1111/medu.14060 31971267

[6] Sonstein SA , Jones CT. Joint task force for clinical trial competency and clinical research professional workforce development. Front Pharmacol. 2018;9:1148. doi: 10.3389/fphar.2018.01148. 30386238 PMC6198073

[7] Johari J , Sahari J , Wahab DA , et al. Difficulty index of examinations and their relation to the achievement of programme outcomes. Procedia - Soc and Behav Sci. 2011;18:71–80. doi: 10.1016/j.sbspro.2011.05.011.

[8] Kocdar S , Karadag N , Sahin MD. Analysis of the difficulty and discrimination indeices of multiple-choice questions according to cognitive levels in an open and distance learning context. Turkish Online J Educ Technol. 2016;15(4):16–24.

[9] Shapiro D , Ryu M , Huie F , Liu Q. Some College, No Degree: A 2019 Snapshot for The Nation and 50 States. Signature Report No 17. Herndon,VA: National Student Clearinghouse; 2019.

[10] Onish K , Karimi DP , Hata J , et al. The badges program: a self-direccted learning guide for residents for conducting research and a successful peer-reviewed publication. MedEdPortal. 2016;12:19443–19453. doi: 10.15766/mep_2374-8265.10443. PMC6464421 31008221

[11] Desmarchelier R , Cary LJ. Toward just and equitable micro-credentials: an Australian perspective. Int J Educ Technol High Educ. 2022;19(25):25. doi: 10.1186/s41239-022-0332-y. 35669714 PMC9155197

